# Feasibility study of DCs/CIKs combined with thoracic radiotherapy for patients with locally advanced or metastatic non-small-cell lung cancer

**DOI:** 10.1186/s13014-016-0635-5

**Published:** 2016-04-21

**Authors:** Luping Zhang, Yanmei Xu, Jie Shen, Feng He, Dan Zhang, Zhengtang Chen, Yuzhong Duan, Jianguo Sun

**Affiliations:** Cancer Institute of PLA, Xinqiao Hospital, Third Military Medical University, Chongqing, 400037 China; Oncology Department, Leshan People’s Hospital, Sichuan, 614000 China

**Keywords:** Dendritic cells, cytokine-induced killer cells, thoracic radiotherapy, non-small cell lung cancer, cytotherapy

## Abstract

**Background:**

The combination of dendritic cells (DCs) and cytokine-induced killer cells (CIKs) can induce the anti-tumor immune response and radiotherapy may promote the activity. We aimed to explore the feasibility of DCs/CIKs combined with thoracic radiotherapy (TRT) for patients with locally advanced or metastatic non-small-cell lung cancer (NSCLC).

**Method:**

In this study, patients with unresectable stage III/IV NSCLC and an Eastern Cooperative Oncology Group performance status (ECOG PS) of 0–2 and previously receiving two or more cycles of platinum-based doublet chemotherapy without disease progression received TRT plus DCs/CIKs or TRT alone until disease progression or unacceptable toxicity. The primary endpoint was median progression-free survival (mPFS). In treatment group, patients received four-cycle autologous DCs/CIKs infusion starting from the 6^th^ fraction of irradiation.

**Results:**

From Jan 13, 2012 to June 30, 2014, 82 patients were enrolled, with 21 patients in treatment group and 61 in control group. The mPFS in treatment group was longer than that in control group (330 days vs 233 days, hazard ratio 0.51, 95 % CI 0.27–1.0, *P* < 0.05), and the objective response rate (ORR) of treatment group (47.6 %) was significantly higher that of control group (24.6 %, *P* < 0.05). There was no significant difference in disease control rate (DCR) and median overall survival (mOS) between two groups (*P* > 0.05). The side effects in treatment group were mild and there was no treatment-related deaths.

**Conclusion:**

The combination of DCs/CIKs with TRT could be a feasible regimen in treating locally advanced or metastatic NSCLC patients. Further investigation of the regimen is warranted.

## Introduction

Lung cancer is the most commonly diagnosed cancer worldwide (1.8 million, 13.0 % of the total), and also a leading cause of cancer death (1.6 million, 19.4 % of the total) [1]. Patients with non-small cell lung cancer (NSCLC) account for more than 80 % of those with lung cancers [2]. Although much progress has been made in the last decade in lung cancer treatment, the overall 5-year survival rate is still less than 20 % [3]. More efforts are needed to improve the prognosis of NSCLC patients.

Thoracic radiotherapy (TRT) plays an irreplaceable role in treating NSCLC patients, especially those with medically inoperable or locally advanced unresectable disease [4]. Accumulating evidences show that TRT may stimulate the anti-tumor immune response [5–8]. Tumor cells killed by irradiation of more than a total dose of 10Gy [9] release tumor antigens that induce numerous immune modulatory molecules [10, 11] and promote tumor-specific effector CD8^+^ T cells via dendritic cell (DC) activation [7]. DCs are the major antigen-presenting cells, and play a central role in regulating and activating anti-tumor immune response [12, 13]. CIKs which express both T cell marker CD3^+^ and NK cell marker CD56^+^ display a strong anti-tumor activity [14]. DCs/CIKs cytotherapy is clinically efficient and can be well tolerated in tumor patients [15, 16]. Based on the hypothesis that DCs/CIKs combined with TRT could benefit the NSCLC patients, we therefore sponsored a phase II clinical trial from January 2012 to June 2014, and explored the efficacy, safety and immunologic effects of DCs/CIKs combined with TRT in patients with NSCLC.

## Patients and methods

### Study design and patients

This prospective single-center, open-label, phase II study was conducted at the Cancer Institute of PLA, Xinqiao Hospital, Third Military Medical University, Chongqing, China. This trial was registered at Chinese Clinical Trial Registry (ChiCTR-TRC-12002369, http://www.chictr.org.cn) and approved by the Ethics Committee of General Logistics Department of PLA, China.

Eligible patients were histologically or cytologically (not including sputum cytology) diagnosed with unresectable stage III or IV advanced NSCLC (according to the 7^th^ edition of the General Rule for Clinical and Pathological Record of Lung Cancer) [17]. All enrolled stage III patients were reluctant to receive or were not suitable for concurrent chemoradiotherapy or radical radiotherapy because of certain conditions, such as huge primary tumors, potential risk of heart failure, respiratory dysfunction and previous chemotherapy in other medical center, etc. Other inclusion criteria included an age of 18 years or older at the time of signing consent form; a life expectancy of 3 months or longer at the registration; an Eastern Cooperative Oncology Group performance status (ECOG PS) of 0–2; adequate function of the liver, kidney, heart and hematopoietic system; two or more cycles of previous platinum-based doublet chemotherapy without disease progression. No previous DCs/CIKs cytotherapy or TRT was allowed. One or more measurable lesions are necessary for therapeutic evaluation based on Response Evaluation Criteria in Solid Tumors (RECIST 1.1) [18]. All study participants provided “written informed consent”. Major exclusion criteria included an acute infection; any autoimmune disease; a history of severe allergic reaction; HIV-positivity; pregnancy or nursing.

**Fig. 1:**
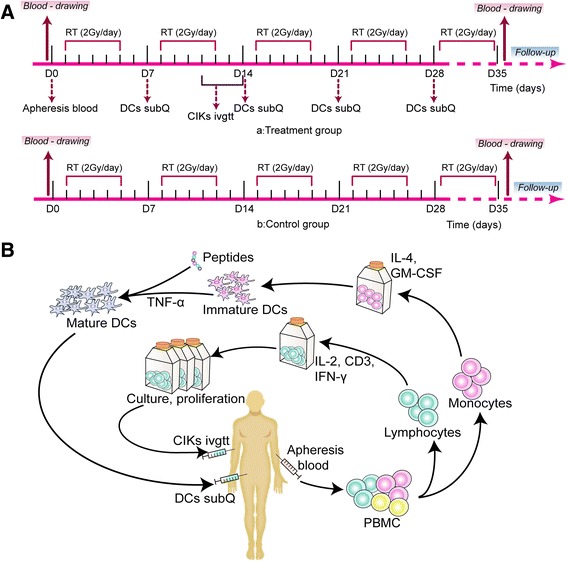
Study design and workflow. **a** The study design of treatment group and control group in clinical trial. **b** The schematic diagram of collection and infusion of autologous DCs/CIKs cells

A block randomization was designed at the beginning, with estimated median progression-free survival (mPFS) of 6 months in control group, one-sided significance level of 0.1 and a power of 0.7. The target sample size was set at 120 patients (1:1), and dropouts were allowed. However, it would take a very long period of time to finish the enrollment because of the high medical cost of DCs/CIKs cytotherapy, which was excluded from medical insurance in China. Therefore, from Jan 13, 2012 to June 30, 2014, enrolled patients were assigned to control group and treatment group at their will instead of randomization. Patients in control group received TRT alone, while the patients in treatment group received TRT in combination with DCs/CIKs cytotherapy that started from the 6^th^ fraction of irradiation (Fig. 1a). The primary endpoint for this clinical trial was mPFS, and the secondary endpoints were objective response rate (ORR), disease control rate (DCR), median overall survival (mOS), PS change and side effects. Immunologic effects were to be explored. After TRT, enrolled patients would continue chemotherapy to reach a standard of 6 cycles in total.

### Preparation of autologous DCs and CIKs

Autologous DCs and CIKs were prepared following the previous studies [19–21] (Fig. 1b). Briefly, peripheral blood mononuclear cells (PBMCs) were isolated by Ficoll-Hypaque gradient density centrifugation, and then cultured in X-VIVO medium for 2 h. The adherent cells were collected for preparing DCs in X-VIVO medium containing granulocyte macrophage colony-stimulating factor (GM-CSF) and interleukin-4 (IL-4). Five days later, tumor necrosis factor-α (TNF-α) and MUC-1 peptide (SAPDTRPAPGSTAPPAHGVT) (GL Biochem, Shanghai, China) were added into DCs culture for another 2 days. For preparing CIKs, non-adherent cells were cultured in X-VIVO medium containing interferon γ (IFN-γ), CD3 monoclonal antibody, and interleukin-2 (IL-2) for 10 days. The immune phenotype markers CD80, CD83, CD86, and HLA-DR for DCs and CD3, CD56 for CIKs were analyzed by flow cytometry. Contamination of bacteria, fungi and endotoxin in all the cultured samples were detected during the course of cell culture.

### DCs/CIKs cytotherapy

At the beginning of the study (day 0), we collected PBMCs from the patients for culturing DCs and CIKs respectively in vitro. Subsequently, over 1 × 10^7^ DCs were injected subcutaneously in the lymph node-rich regions (bilateral axillary or inguinal region) on days 7, 14, 21, and 28. Over 1 × 10^9^ CIKs in 100 mL of of normal saline (NS) (0.9 %) were infused intravenously once a day for 4 consecutive days from day 11 to day 14 (Fig. 1a).

### TRT regimens

The interval between chemotherapy and enrollment was no less than 14 days. TRT including three-dimensional conformal radiotherapy (3D-CRT) or intensity-modulated radiotherapy (IMRT) was adopted according to NCCN guideline for patients with advanced NSCLC. Contour delineation and radiotherapy plan was designed and confirmed by the professional radiation oncologist. TRT was delivered at 2 Gy per fraction, 5 fractions per week, to a total dose of 60–66 Gy at planning gross tumor volume (pGTV) in 6–7 weeks. All plans were performed with the support of four-dimensional chest CT. The normal lungs received a limited radiation according to NCCN guidelines.

### Assessment of clinical outcomes

According to RECIST 1.1 [18], the treatment efficacy was classified as complete response (CR), partial response (PR), stable disease (SD), and progression disease (PD). The ORR was defined as the percentage of patients with CR or PR, and DCR was defined as the percentage of patients with CR, or PR, or SD. mPFS was defined as the median time scale from enrollment to disease progression, while mOS was the median time scale from first treatment to death. The follow-up was performed at the 1^st^ and 3^rd^ month after TRT, and then every 3 months for the first year, and every 6 months thereafter. Routine follow-up assessments included physical examinations, vital signs, computed tomographic scans (CT), and laboratory tests.

### Assessment of immunologic effects

Blood-drawing from participants was performed on day 0 and within a week after TRT (Fig. 1a). Cytokines (IL-2, IFN-γ) in serum were detected by enzyme-linked immunosorbent assay (ELISA) (R&D Systems, MN, USA) following the manufacturer’s instruction. For assay of T cell populations and NK cells, 100 μL of EDTA anticoagulant blood samples were stained with corresponding antibodies (BD Bioscience), namely, anti-CD3^+^, CD4^+^ and CD8^+^ for T cells, anti-CD3^+^ and CD56^+^ for NK cells, in darkness for 20 min. Then, erythrocyte lysis buffer was added. After being vortexed for 15 s and incubated at room temperature for 5 min, the samples were centrifuged to remove the supernatant and washed with PBS. After being re-suspended with staining buffer, the samples were analyzed on the BD Aria flow cytometer (BD Bioscience).

### PS and side effects

Adverse effects, such as insomnia, anorexia, fever, skin rash, and joint pain, were monitored and were observed once a week during the therapy and once a month during the follow-up. PS was evaluated on day 0 of the study and within a week after the TRT.

### Statistical analysis

The measurement data were expressed as mean ± standard deviation $\left(\overline{\mathrm{x}}\pm \mathrm{s}\right)$ and analyzed with the independent Student *t* test. The enumeration data were analyzed using *χ*^*2*^ test. Kaplan-Meier curves with the log-rank test were used to estimate mPFS and mOS. Hazard ratio (HR) and 95 % CI were also calculated by Cox proportional hazard regression models. *P* < 0.05 was considered statistical significance.

## Results

### Patient characteristics

**Fig. 2:**
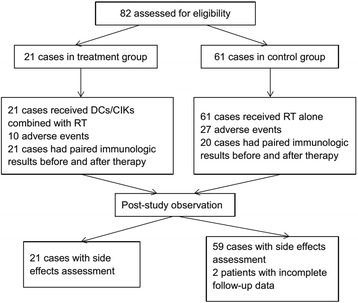
The profile of clinical trial

From January 13, 2012 to June 30, 2014, a total of 82 patients with locally advanced or metastatic NSCLC were enrolled, with 21 patients in treatment group and 61 in control group (Fig. 2). Clinicopathological characteristics such as age, gender, PS, clinical stage of tumor, previous systemic chemotherapy, pathological type and PS in treatment and control groups were analyzed. None of them showed significant differences (Table 1, *P* > 0.05), which meant a nearly identical baseline between the two groups.

**Table 1:** Baseline characteristics of two groups

Characteristics	Treatment group	Control group	*P* value
Number	21	61	
Age (years)			
Mean (Range)	56.6 (32–74)	56.4 (31–74)	0.95
Gender			
Male	19 (90.5 %)	53 (86.9 %)	
Female	2 (9.5 %)	8 (13.1 %)	0.73
Clinical stage			
III	10 (47.6 %)	37 (60.7 %)	
IV	11 (52.4 %)	24 (39.3 %)	0.30
Tumor histology			
Adenocarcinoma	8 (38.1 %)	26 (42.6 %)	
Squamous carcinoma	13 (61.9 %)	35 (57.4 %)	0.72
Cycles of previous chemotherapy	2.9 ± 0.7	3.1 ± 0.8	0.53
PS score	0.4 ± 0.6	0.6 ± 0.7	0.35

### Clinical outcomes

**Fig. 3:**
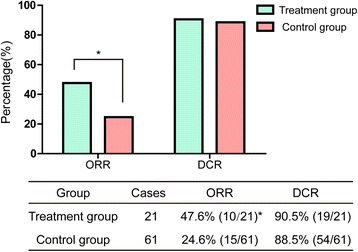
The short-term clinical effects. *ORR is significantly higher in treatment group (*P* < 0.05)

The median follow-up time in treatment and control groups was 339 and 393 days, respectively. 0 CR, 10 PR, 9 SD and 2 PD were found in treatment group, and 0 CR, 15 PR, 39 SD and 7 PD were found in control group. ORR in treatment group is higher than that in control group (47.6 % vs. 24.6 %, *P =* 0.04) (Fig. 3). However, no obvious difference in DCR was observed between the two groups (90.5 % vs. 88.5 %, *P* = 0.767).

**Fig. 4:**
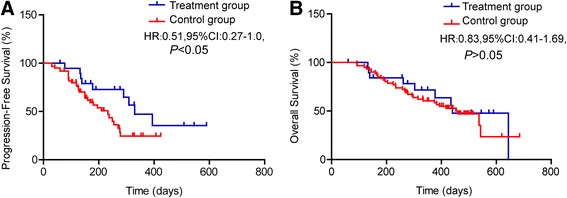
Kaplan-Meier curves of mPFS and mOS. **a**. Compared with control group, mPFS in treatment group is significantly longer (*P* < 0.05). **b**. No significant mOS difference between treatment group and control group (*P* > 0.05)

As for long-term evaluation, the mPFS of treatment group (330 days) was significantly longer than that of control group (233 days) (HR 0.51, 95 % CI 0.27–1.0, *P* = 0.0483). However, there was no significant difference in mOS between treatment group (400 days) and control group (460 days) (HR 0.83, 95 % CI 0.41–1.69, *P* = 0.606) (Fig. 4).

### Immunologic response

Among the 61 patients in control group, complete immunologic results were obtained in only 20 cases before and after TRT. There was a lack of some medical materials in the rest patients because of their refusal to draw blood and the delayed follow-up, and some other reasons. These 20 cases were analyzed by assessing the baseline (Table 2) and immunologic effects. The results of cytokines (IL-2, IFN-γ), T cell populations and NK cells were analyzed. The serum levels of IL-2 and IFN-γ did not differ significantly between the two groups both before and after the TRT (Table 3, *P* > 0.05). Moreover, there were no obvious changes in the percentage of CD3^+^, CD3^+^CD4^+^, CD3^+^CD8^+^, CD4^+^/CD8^+^ T cell ratio and CD3^−^CD56^+^ NK cells before and after TRT in treatment group (*P* > 0.05). However, it should be noted that there was a decrease in CD4^+^/CD8^+^ T cell ratio after TRT in control group, with a *P* value close to 0.05 (Table 3, *P* = 0.08).

**Table 2:** Baseline characteristics of two groups in immunologic response

Characteristics	Treatment group	Control group	*P* value
Number	21	20	
Age (years)			
Mean (Range)	56.6 (32–74)	54.3 (39–68)	0.48
Gender			
Male	19 (90.5 %)	17 (85 %)	
Female	2 (9.5 %)	3 (15 %)	0.66
Clinical stage			
III	10 (47.6 %)	10 (50 %)	
IV	11 (52.4 %)	10 (50 %)	0.88
Tumor histology			
Adenocarcinoma	8 (38.1 %)	10 (50 %)	
Squamous carcinoma	13 (61.9 %)	10 (50 %)	0.44
Cycles of previous chemotherapy	2.9 ± 0.7	2.4 ± 0.5	0.06
PS score	0.4 ± 0.6	0.5 ± 0.6	0.91

**Table 3:** Immunology response of patients $\left(\overline{x}\pm \mathrm{s}\right)$

Group	CD3^+^(%)	CD3^+^CD4^+^(%)	CD3^+^CD8^+^(%)	CD4^+^/CD8^+^	CD3^−^CD56^+^(%)	IL-2 (ng/L)	IFN-r (pg/mL)
Treatment group							
Pre-treatment	62.16 ± 13.62	33.64 ± 10.05	25.86 ± 10.30	1.55 ± 0.88	17.83 ± 9.04	330.42 ± 79.25	575.85 ± 179.85
Post-treatment	66.34 ± 13.65	34.63 ± 13.28	29.73 ± 11.14	1.45 ± 0.97	17.31 ± 9.50	330.94 ± 66.12	567.12 ± 151.64
* P* value	0.3	0.716	0.119	0.684	0.806	0.979	0.831
Control group							
Pre-treatment	68.70 ± 15.48	39.48 ± 12.76	27.30 ± 8.79	1.57 ± 0.67	10.25 ± 6.12	358.37 ± 49.00	491.19 ± 60.00
Post-treatment	70.43 ± 19.67	33.6471 ± 18.02	33.65 ± 17.19	1.27 ± 0.96	8.52 ± 6.52	376.09 ± 44.44	507.32 ± 59.87
* P* value	0.705	0.051	0.077	0.08	0.378	0.186	0.481

### PS and side effects

At the beginning of the study, the PS in treatment and control groups was 0.4 ± 0.6 and 0.6 ± 0.7, respectively (Table 1). At the end of TRT, the PS in treatment and control groups were 0.9 ± 0.8 and 1.4 ± 0.6, respectively. Little PS increase was found in treatment group after TRT (0.48 ± 0.7). However, obvious PS increase was recorded in control group (0.9 ± 0.7). The PS increase in treatment group was significantly lower than that in control group (*P* = 0.018, Table 4).

**Table 4:** PS and side effects

Characteristics	Treatment group (*n* = 21)			Control group (*n* = 59)		
	Grade 1–2	Grade 3	Grade 4	Grade 1–2	Grade 3	Grade 4
Fever	5 (23.8 %)	0	0	13 (22.0 %)	0	0
Anorexia	3 (14.3 %)	0	0	6 (10.2 %)	0	0
Allergy	1 (4.8 %)	0	0	0	0	0
Nausea, vomiting	3 (14.3 %)	0	0	9 (15.3 %)	0	0
Heart function	0	0	0	0	0	0
Liver function	0	0	0	0	0	0
Renal function	0	0	0	0	0	0
Myelosuppression	2 (9.5 %)	0	0	8 (13.6 %)	0	0
Radiation pneumonitis	4 (19.0 %)	3(14.3 %)	0	11 (18.6 %)	9(15.3 %)	0
PS change after TRT	0.48 ± 0.7*			0.9 ± 0.7		

*PS change is significantly better than control group (*P* < 0.05)

Side effects were assessed in all the 21 cases in treatment group, and 59 of 61 cases in control group, with incomplete follow-up information in 2 cases. The functions of the liver, kidney and heart of all the participants remained normal at the end of the TRT treatment. The most commen side effects were fever, anorexia, nausea, vomiting, myelosuppression, and radiation pneumonitis (Table 3). Most of them were at level I ~ II, except radiation pneumonitis. Radiation pneumonitis with grade 3 was observed in 3 patients in treatment group (14.3 %), and 9 patients in control group (15.3 %). All patients recovered after suitable treatment within 2 months. There were no cases with grade 4 radiation pneumonitis and treatment-related deaths.

## Discussion

Cancer cytotherapy is a novel therapeutic approach with great potential [22–24]. Since the report of the first DCs-based cancer vaccine clinical trial in 1995 [25], a lot of trials have been designed and conducted [26, 27]. In 2010, Food and Drug Administration (FDA) approved the first DCs-based vaccine Provenge for the treatment of advanced prostate cancer [23, 28]. Additionally, the cytotoxic and regulatory anti-tumor effects of CIKs are also attractive and promising. The combination of DCs with CIKs is a viable adoptive cytotherapy with a strong anti-tumor effect [29, 30]. It was shown that irradiation enhanced MHC I expression, and changed the tumor microenvironment to boost greater infiltration of immune-effector cells [31–33]. Tumor cells killed by irradiation released tumor antigens which were presented by ectopic DCs [10]. Both preclinical and clinical researches proved that radiotherapy combined with cytotherapy elicited greater anti-tumor response [34, 35].

As for the clinical outcomes of our study, a longer mPFS was observed in treatment group than in control group (330 days vs 233 days, *P* < 0.05), and ORR was higher in treatment group (47.6 % vs 24.6 %, *P* < 0.05). Although there was no significant difference in DCR and mOS between the two groups (*P* > 0.05), the positive results in mPFS and ORR were still encouraging. Thus, patients treated with DCs/CIKs combined with TRT had a better clinical benefit. In the present study, we started DCs/CIKs cytotherapy from the 6^th^ fraction of TRT to release enough tumor antigens. Our results validate the hypothesis that tumor antigens released by TRT could enhance tumor-specific killing via ectopic DCs/CIKs infusion.

For safety analysis, during the combination therapy of DCs/CIKs and TRT, a majority of side effects were mild, tolerant and similar to TRT alone. No new safety signals were identified, and no treatment-related deaths occurred. In addition, we found a significant PS increase after TRT in control group (*P* < 0.05). Nevertheless, there was a minor PS increase in treatment group (*P* > 0.05). It suggests that combined cytotherapy improves the PS for advanced patients receiving TRT. Thus, DCs/CIKs in combination with TRT shows a good safety profile.

Cancer patients often suffer from immune deficiency, including a decrease in CD4^+^/CD8^+^ T cell ratio, especially during a long period of systemic chemotherapy [36]. In the present study, we found that there was a tendency of a decrease in CD4^+^/CD8^+^ T cell ratio after TRT in control group (*P* = 0.08) instead of in treatment group (Table 2). Thus, a reasonable explanation could be that radical TRT with conventional fractionation causes immune suppression in control group, and DCs/CIKs cytotherapy partially rescues immune suppression induced by TRT in treatment group.

Meanwhile, the current study detected other cytokines, such as IL-2 and IFN-γ in peripheral blood, which were supposed to play critical roles in specific immunological effects and promoting innate and adaptive immune responses [37]. The serum levels of IL-2 and IFN-γ did not change significantly after TRT in both groups (*P* > 0.05). Since the immune response is very complex in DC/CIK combined with TRT, further research is needed to reveal cytokine activity in the future.

Given that irradiation-mediated immune responses alter the tumor micro-environment, more and more researches have explored that local radiation combined with CTLA-4 blockade [38] or PD-L1 blockade [39] could promote anti-tumor immunity. Our results also suggest that the combination of cytotherapy with TRT is a novel feasible application. It shows better clinical benefit, a good tolerance, minor PS change, and promotes the immunity to some extent. However, further studies are needed with larger sample sizes. In addition, due to the lack of randomization and thus possible bias (e.g. more wealth, better education, better supportive care in treatment group), activity needs to be further evaluated in a properly designed randomized trial. Standardized treatment schedule and detailed mechanism of DCs/CIKs combined with TRT should be elucidated in the ongoing research.

## Conclusions

Our study confirms the efficiency and safety of the combination of DCs/CIKs cytotherapy with TRT in advanced NSCLC. Indeed, this novel strategy enhances immunity, improves ORR, prolongs mPFS, and barely changes PS, with no severe treatment-related side effects. It is therefore a feasible regimen for patients with advanced NSCLC.
